# Double Diapering Ineffectiveness in Avoiding Adduction and Extension in Newborns Hips

**DOI:** 10.3390/children8030179

**Published:** 2021-02-26

**Authors:** Maurizio De Pellegrin, Chiara Maria Damia, Lorenzo Marcucci, Desiree Moharamzadeh

**Affiliations:** 1 Pediatric Orthopedic Unit, San Raffaele Hospital (IRCCS Ospedale San Raffaele), 20132 Milan, Italy; depellegrin.maurizio@hsr.it (M.D.P.); lore.marcucci93@gmail.com (L.M.); 2 Residency Program Pediatrics, Vita-Salute San Raffaele University, 20132 Milan, Italy; chiaradamia86@gmail.com; 3 Department of Orthopedic and Traumatology, San Raffaele Hospital, 20132 Milan, Italy

**Keywords:** douple diapering, neonatal hip, DDH prevention, hip positioning, hip extension, hip adduction

## Abstract

Hip flexion and abduction is fundamental for developmental dysplasia of the hip (DDH) treatment. At present, double diaper treatment has been inappropriately adopted when DDH is suspected. The aim of this study was to verify whether double diapers influence a newborn’s hip position. Here, we studied 50 children (23 female; 27 male; average age 62.33 ± 20.50 days; average birth weight 3230 ± 447 g) with type I hips according to Graf. At the same time of the ultrasound (US) examination, the following hip positions were measured using a manual protractor: (1) spontaneous position, supine on the outpatient bed without a diaper; (2) spontaneous position, with a double diaper; and (3) squatting position on the caretakers’ side. Statistical analysis was performed with a *t*-test to compare between (1) the spontaneous position without a diaper and with double diapers; (2) the spontaneous position with double diapers as well as the squatting position on the caretakers’ side with a diaper. The comparison between the hip position without diaper and with double diapers was statistically not significant for all measurements, i.e., right hip flexion (*p* < 0.33), left hip flexion (*p* < 0.34), and right and left hip abduction (*p* < 0.87). The comparison between the hip position with double diapers and on the caretakers’ side was statistically significant for all measurements, i.e., right hip flexion (*p* < 0.001), left hip flexion (*p* < 0.001) and right and left hip abduction (*p* < 0.001). We found that the use of double diapers did not affect hip position, while the position formed on the caretaker’s side shows favorable influence.

## 1. Introduction

The femoral head and the acetabulum mutually influence one another’s growth and evolution starting from the prenatal period. The natural fetal position, also known as the “human position” according to Salter, refers to very flexed and moderately abducted hips. The physiological development of the hip during growth is determined by the centering of the femoral head in the acetabulum, and this is guaranteed by adequate degrees of flexion and abduction of the hips. It is known that the African populations, who culturally keep newborns in this position [1], do not observe hip dysplasia as a newborn and adult disease. On the other hand, it is well known that in countries with a cold climate, such as in Lapland, Northern China, Canada, and Japan, where for climatic reasons infants are swaddled with their lower limbs straight (and therefore with hips in extension and adduction), the risk of hip dysplasia is increased [1,2]. After a public awareness campaign was developed to eliminate these traditions, the incidence of hip dysplasia in infants dropped dramatically [2]. All treatments, past [3] and present [2,4,5,6,7], for both hip dysplasia prevention and management, point out the central concept of positioning the hips flexed and abducted to avoid opposite positions such extension and adduction.

Some health care providers identify the use of double diapers as a simple system to obtain the desired position for the correct development of the hips. In fact, although the technique is not scientifically based, double diapers are often recommended by pediatricians as a first therapeutic step during the waiting period before the instrumental procedure, which currently is the ultrasound (US) examination. It is also recommended as a treatment in cases of limited hip abduction, regardless of the US findings, or as a therapeutic alternative to other more invasive abduction splinting devices. This therapeutic indication is widespread and still suggested. Teanby reported that up to 19.3% of the population in some European countries has been treated with double diapers [8,9].

The introduction of US examination as a screening technique of developmental dysplasia of the hip (DDH) has further refined the diagnosis of dysplasia, allowing the identification of even modest alterations of the acetabulum and introducing the concept of spontaneous correction. In these hips, the double diaper treatment has been widely used as well.

In literature, double or triple diaper treatment for the management and prevention of DDH has always been mentioned, despite the demonstrated doubtful utility [10,11]. The aim of this study is therefore to evaluate whether the double diaper treatment is able to modify the spontaneous position of the newborn’s hips and avoid adduction and extension. 

## 2. Materials and Methods

Data were collected of 50 children (23 females and 27 males), who were consecutively referred to our DDH dedicated outpatient clinic for a clinical ultrasound evaluation of the hips. The clinical history of all newborns was evaluated, with regards to the fetus position during pregnancy and uterus postural anomalies, as well as the fetal anomalies diagnosed using the US examination during pregnancy, twin pregnancy, and weight at birth. The clinical examination included the evaluation of the hips regarding spontaneous posture, limitation of abduction, and the presence of clinical signs of dysplasia (Ortolani and Barlow). All newborns then underwent an US examination of the hips according to Graf [12] performed by the same operator who is certified for this method.

**Figure 1:**
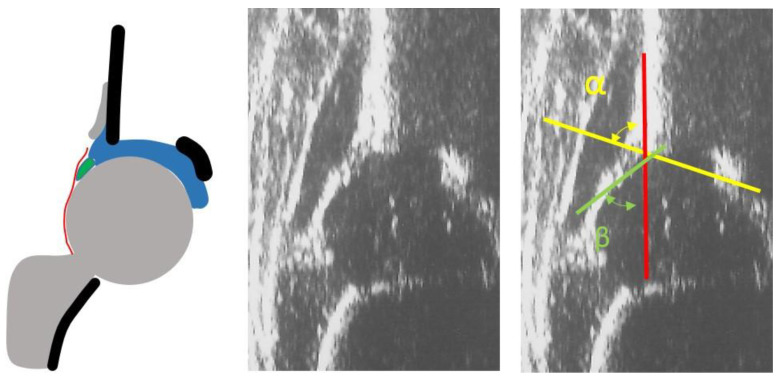
Normal newborn hip. Ultrasound (US) and diagram showing a type I according to Graf.

Inclusion criteria were as follows:

(i) single pregnancy with cephalic presentation and physiological postnatal development
(ii) no functional limitations of the hips at clinical examination, particularly those of abduction
(iii) normal range weight
(iv) infants younger than 3 months
(v) infants with type I hips at the US examination according to Graf classification (Figure 1)

Exclusion criteria were:

(i) twins or non-cephalic presentation in pregnancy (breech, transverse) and abnormal postnatal development
(ii) infants who presented with clinical signs of dysplasia or functional limitations of the hips, particularly abduction
(iii) premature or non-normal weight newborns
(iv) infants older than 3 months
(v) infants with non-Type I hips at US examination according to Graf classification

Average age was 62.33 days (SD ± 20.50 days) and average birth weight was 3230 g (SD ± 447 g). The newborns were measured in the spontaneous position of the hips with a manual protractor [13]. The measurements were all achieved after the US examination in 3 steps: (1) supine, in spontaneous position on the outpatient bed without a diaper; (2) supine, in spontaneous position on the outpatient bed with double diapers; (3) in a squatting position on the caretakers’ side, wearing a single diaper.

**Figure 2:**
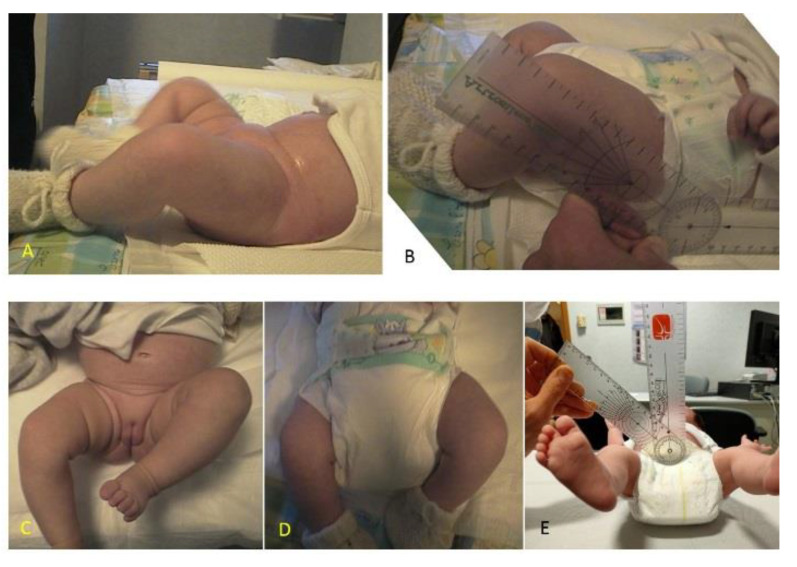
Hip position in a 2-month old infant: (**A**) evaluation of hip flexion without diaper; (**B**) with double diapers. Measurements with a of hip flexion angle with protractor: (**C**) evaluation of hip abduction without diaper; (**D**) with double diapers; (**E**) measurement of abduction with protractor.

**Figure 3:**
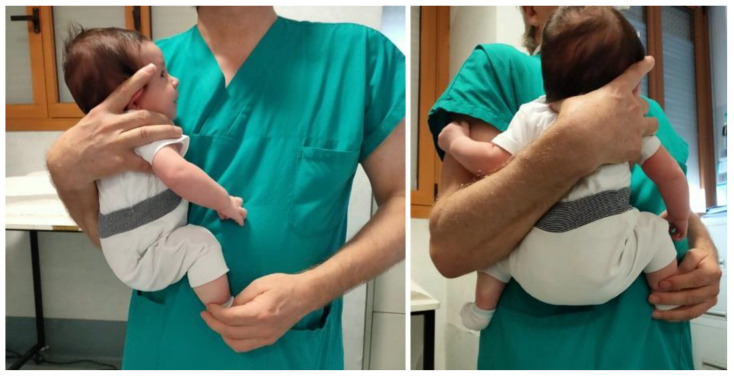
Hip position in a 2-month old infant when held on the caretakers’ side. Flexion is approximately 100° and abduction 60°.

A statistical analysis was performed with a *t*-test in order to compare between (1) the spontaneous position without a diaper and with double diapers, and (2) the spontaneous position with double diapers as well as the squatting position on the caretakers’ side with a diaper (Figure 2 and Figure 3).

## 3. Results

Flexion and abduction measurements data of both the hips (average values and standard deviation, SD) in the spontaneous supine position of the infant on the outpatient bed without diaper, in the spontaneous position of the infant after application of double diapers, and in the squatting position on the caretakers’ side with a diaper, are shown in Table 1 and Table 2. The data of the statistical analysis conducted by a *t*-test for the comparison between the spontaneous position of the hips without a diaper and the spontaneous position with double diapers were statistically not significant for all measurements, i.e., right hip flexion (*p* < 0.33), left hip flexion (*p* < 0.33), right and left hip abduction (*p* < 0.87). The comparison between the spontaneous position of the hips with double diapers and the squatting position on the caretakers’ side with a diaper were statistically significant (*p* < 0.001) for all measurements taken, namely for flexion and for abduction of both the right hip and the left hip (Table 1 and Table 2).

No statistically significant difference has been reported in the hips position with double diapers and without diapers, while there is a statistically significant difference in the position of the hips of infants held on the caretakers’ side compared to infants with double diapers. These do not affect the position of the hips, while the position on the caretakers’ side does.

## 4. Discussion

**Figure 4:**
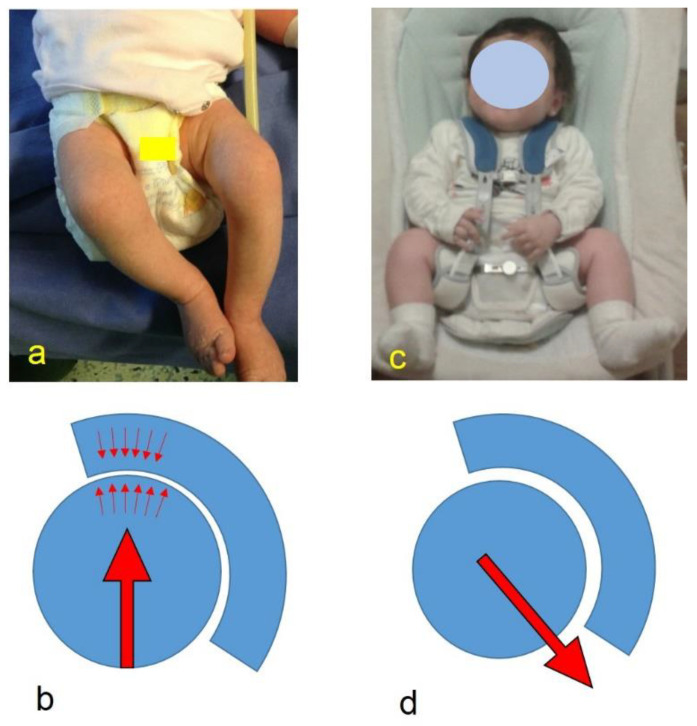
(**a**) Spontaneous hip position with extended and adducted hips; (**b**) diagram to note the force vector’s direction (arrow) and the head’s pressure on the acetabulum (small arrows); (**c**) 2-month-old infant treated with harness for developmental dysplasia of the hip (DDH), with hip flexed and abducted; (**d**) diagram to note the modified direction of the force vector.

At the present date, once DDH is diagnosed with an US examination, its treatment requires a specific hip position in flexion and abduction [4,5]. Flexion is known to be effective when approximately 100° is reached, given that in this position, the pressure on the acetabular region decreases and the dislocating strength of the hamstring muscles is reduced (Figure 4). The abduction favorably centers the femoral head in the acetabulum, but the concept of a “safe zone”, already expressed in 1976 by Ramsey, must be observed, as it takes in consideration the danger of maximum abduction (“frog” position) on the femoral head vascular supply. Therefore, abduction must not exceed 50–60° [14]. In all the prevention strategies, the concepts expressed above regarding hip position are always considered; adduction and extension must be avoided. In 1959, Judet and Gielis specifically proposed placing the legs in abduction for DDH as a prevention measure for all newborns until 4 months of age. In the past, also in Scandinavian countries, if a clinical suspect of DDH was present, infants’ hips were flexed and abducted with the Frejka splint [7]. In Italy, the studies of the pediatrician Marino Ortolani are well known. Ortolani, after identifying the worldwide clinical sign, in an epidemiologically endemic region for DDH, recommended hip flexion and abduction even where the “clunk” was absent. After this experience, the attitude of aiding the development of the hips by regular wide diapering with hips in abduction of all presumably healthy hips (essentially all children) has spread not only in Italy but also elsewhere. The abduction and flexion were obtained by means of a starched abduction towel in order to direct possible cases of dysplasia towards normal development. In particular, Klisic et al. [2,6] have precisely described the use of a suitably folded baby package, which correctly protects the hips (helping to prevent DDH) by maintaining them in mild flexion and mild abduction during the neonatal period. The study reported a significant decrease in the prevalence of congenital dislocation of the hip (from 1.3% to 0.7%) 4 years after the introduction of the Klisic method [6].

The introduction of soft industrial diapers has considerably changed the effectiveness of the Klisic baby packages. However, the concept was maintained historically. As a matter of fact, this is the basis from which the advice of wearing double diapers as a preventive measure for DDH derives. The use of double diapers is not recommended by the American Academy of Pediatrics, which defined this therapeutic intervention as an inappropriate one and as a cause of delay in treatment (use of adequate devices) if positioned in hip dislocations [15]. The Canadian Task Force on Preventive Health Care also stated the absence of clear evidence to support the use of double or triple diapers [16]. A further study by Stephen K. Storer and David L. Skaggs confirmed no evidence of improvement of hip dysplasia when compared with non-intervention [17]. Moreover, double diapers are sometimes inappropriately seen as an alternative solution to the various abduction splints, mostly because it is considered psychologically more acceptable (Figure 4). However, no study has yet been performed where the hip position of a newborn with double diapering is objectively evaluated and in which clearly states its non-recommendation for therapeutic uses. When a DDH is diagnosed, the same problems already reported by other authors mentioned above [15,16,17] are also present.

**Figure 5:**
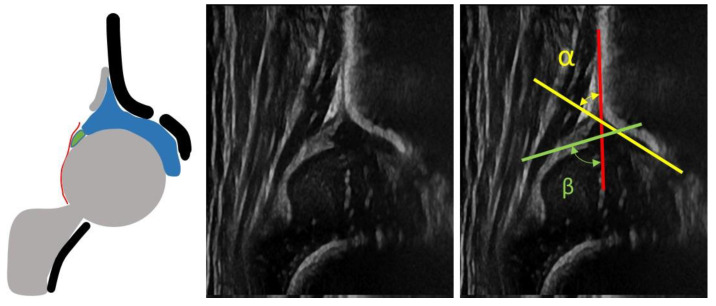
US image and diagram of a physiologically immature hip of a 6-week old infant. Type IIa hip according to Graf.

The approach to this disease has dramatically changed since the introduction of US examination of the hips, used initially as a diagnostic tool and subsequently as a gold standard screening method for DDH [18]. In the past, only hip dislocations were detected and the reported incidence was 0.13%; since the introduction of US as a precise diagnostic tool that is able to show even minimal alterations of the acetabulum, the reported incidence has increased to 1.6% among the general population [19]. The US examination introduced with type IIa hips (Figure 5) the concept of physiological immaturity of the hip, which is not an expression of pathology; the hips with lower bony coverage than normal at birth (alpha angle ≥ 50° and < 60°) show in 98% of cases a spontaneous evolution towards normal bone coverage [20]. In addition, in these cases, the opinion of recommending double diapers is widespread in some European countries.

Thus, regarding both treatment and prevention of DDH, the real effectiveness of double diapers must still be verified, with particular attention to the real modification it can determine with respect to the position of the hip, particularly in avoiding hip adduction and extension. It is known that infants have an average hip flexion contracture of 28° at birth. The hip flexion contracture decreases to 19° at 6 weeks and to 7° at 3 months of age [21]. The measurements observed in our data without a diaper and with a double diaper do not differ significantly from these degrees of flexion. In other words, the measurement of the hips with double diapers has shown that this procedure is neither able to change the position of the hips nor guarantee the degrees of flexion and abduction necessary to achieve the correct acetabular maturation and also to avoid adduction. Our data were collected in healthy infants without functional limitations with symmetrical hip range. The ineffectiveness of double diapers is further validated in hips that already have a functional limitation, as no improvement is highlighted. Instead, the position on the caretakers’ side with flexed and abducted hips is able to help in avoiding adduction and extension of the lower limbs. Obviously the requirement is that this position must be maintained constantly, which is difficult to achieve in daily life. However, the simple advice to hold infants on the side is the main idea of this study. In this regard, the current transport trends for newborns such as baby carriers and baby wrap carriers, which perhaps unknowingly respect the position of the hips described above, may be helpful and recommended for the prevention of DDH. Indeed, some recent swaddling devices must not be recommended as they imply an unfavorable hip position.

## 5. Conclusions

We can conclude that double diapering does not influence a newborn’s hip position; the study particularly demonstrated its ineffectiveness in preventing hip adduction and extension, and it was shown to be an unfavorable position for hip development. Instead, the position on the caretakers’ side or in baby carriers and baby wrap carriers, which places the hips in flexion and abduction, will favorably influence hip development.

## Figures and Tables

**Table 1:** Flexion and abduction degrees measurement of the right (R) and left (L) hips with standard deviation (SD) without diaper and with double diapers.

Hips Position	Without Diaper	Double Diapers	*p* *
R Flexion Average (SD)	25.25° (18.46°)	27.5° (21.4°)	0.33
L Flexion Average (SD)	25° (18.57°)	28.64° (22.99°)	0.34
R Abduction Average (SD)	24° (8.83°)	35.06° (26.85°)	0.87
L Abduction Average (SD)	23.5° (8.75°)	34.39° (25.12°)	0.87

* *p*: Student’s *t*-test

**Table 2:** Flexion and abduction degrees measurement of the right (R) and left (L) hip with standard deviation (SD) with double diapers and on the caretakers’ side.

Hips Position	Double Diapers	Caretakers’ Side	*p* *
R Flexion Average (SD)	27.5° (21.4°)	90.74° (9.97°)	< 0.001
L Flexion Average (SD)	28.64° (22.99°)	90° (10.38°)	< 0.001
R Abduction Average (SD)	35.06° (26.85°)	54.44° (10.13°)	< 0.001
L Abduction Average (SD)	34.39° (25.12°)	54.26° (9.87°)	< 0.001

* *p*: Student’s *t*-test

## Data Availability

The datasets used and/or analyzed during the current study are available from the corresponding author on reasonable request.
